# dCATCH-Seq: improved sequencing of large continuous genomic targets with double-hybridization

**DOI:** 10.1186/s12864-017-4159-7

**Published:** 2017-10-23

**Authors:** Yanfeng Zhang, Jun Song, Kenneth Day, Devin Absher

**Affiliations:** 1 HudsonAlpha Institute for Biotechnology, Huntsville, USA; 2 Ubiquity Genomics Inc., Huntsville, USA

**Keywords:** Targeted sequencing, Mhc, Hla, DNA methylation, Hybridization, Bac

## Abstract

**Background:**

Targeted sequencing is a powerful tool with broad application in both basic and translational sciences. Relatively low on-target rates for most current targeted sequencing studies influence the required coverage and data quality for subsequent applications.

**Results:**

We present an improved targeted sequencing method that uses two rounds of in solution hybridization with probes synthesized from genomic clone templates, termed dCATCH-Seq. Independent captures of two large continuous genomic regions across three cell types within the human major histocompatibility complex (MHC) that spans ~3.5 Mb and a ~250 kb region on chromosome 11 demonstrated that dCATCH-Seq was highly reproducible with ~95% capture specificity. Comprehensive analyses of sequencing data generated using the dCATCH-Seq approach also showed high accuracy in the detection of genetic variants and HLA typing. The double hybridization capture approach can also be coupled with bisulfite sequencing for DNA methylation profiling of both CpG and non-CpG sites.

**Conclusions:**

Altogether, dCATCH-Seq is a powerful and scalable targeted sequencing approach to investigate both genetic and epigenetic features.

**Electronic supplementary material:**

The online version of this article (10.1186/s12864-017-4159-7) contains supplementary material, which is available to authorized users.

## Background

The primary design of targeted sequencing is to capture genetic variants within intended regions [1]. Some targets, such as exome capture, has been broadly used in both basic and translational research that includes characterization of genetic diversity and demographic history in human populations [2, 3], identification of etiological variants [4], cross-species genome comparisons [5], and even phylogenetic estimation [6].

There are three main techniques [7] for targeted enrichment of DNA sequences that include hybridization-based capture either in solution or on a solid support, e.g., TruSeq (Illumina), SureSelect (Agilent), and SeqCap (Roche NimbleGen) platforms [8–10], selective circularization, and PCR amplification. In principle, targeted sequencing approaches should efficiently capture DNA molecules within the intended genomic regions with little to no sequences outside these regions of interest. However, most of current targeted sequencing studies, including commercialized whole-exome sequencing, generally show a target specificity in a range from 40% to 80%, and rarely approach 90%, regardless of probes used in hybrid selection [9–15]. Although some off-target regions neighboring intended targets such as splice sites and intron edges in exome capture “splash”, could be informative [16], the capture of the non-target regions at different levels may substantially impact data quality and adequate coverage across the intended targets, and eventually require more sequencing costs. Some strategies in measuring target efficiency have also been developed, e.g., SeqCap qPCR kit (Roche) and Multipoint Test for Targeted-enrichment Efficiency (MTTE) [17].

We previously reported the clone adapted template capture hybridization sequencing (CATCH-Seq) procedure that synthesizes probe sets from a pool of selected BAC or fosmid DNA templates for in solution hybrid capture [13]. We have employed an updated target capture strategy to improve on target capture efficiency of large genomic regions by two rounds of hybridization in solution that we term double CATCH-Seq (dCATCH-Seq). To evaluate the performance of this procedure, we independently tested two large continuous genomic regions, including the entire major histocompatibility complex (MHC), covering a size of 3.5 Mb, using a custom probe set generated from a pool of 140 reference BAC DNAs. Our updated approach provides a more efficient alternative to the previously reported approach, and demonstrates the feasibility of capturing large and diverse genomic regions that enables new applications such as high resolution HLA typing.

## Results

### Double versus single hybridization capture

**Fig. 1:**
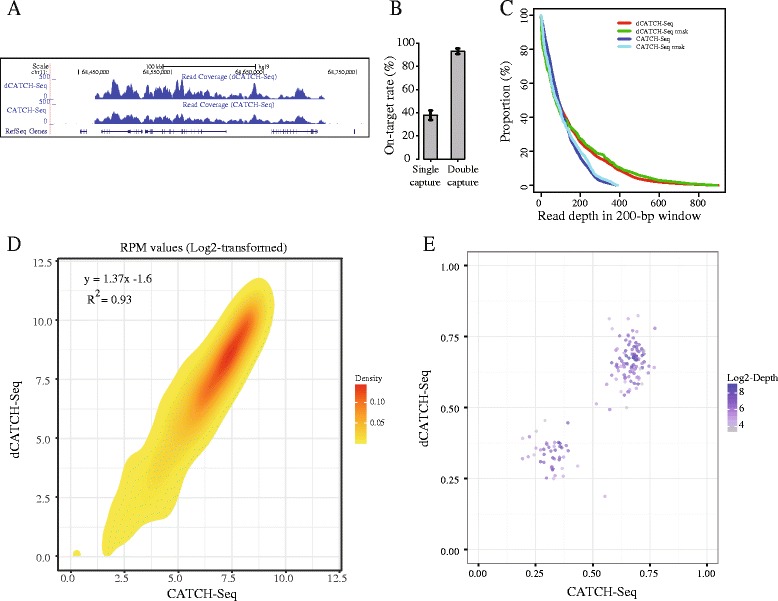
Comparison of double versus single hybridization capture approach. **a** Wiggle plot showing the coverage across an intended region on chr11 (chr11:68,467,857–68,716,491, hg19). Two tracks shown in dark blue are the read coverage in 50-bp bins for dCATCH-Seq and CATCH-Seq approaches (**b**) Barplot of on-target rates for single and double captures. Error bars are the standard deviations from technical replicates (*n* ≥ 5). **c** Plot of the proportion of reads against the read depth based on coverage in 200 bp non-overlapping windows across the intended region on chr11. Rmsk, repeat maskering, means plot based on the repeat masked read depth. **d** Density plot showing the comparison of read depth normalized by using log2-transformed RPM values between CATCH-seq and dCATCH-seq data. The linear regression equation and Pearson’s Correlation coefficient are shown in the left top corner. **e** Scatterplot showing the comparison of read depth (log2-transformed) for heterozygous variants called from CATCH-seq (x-axis) and dCATCH-seq (y-axis)

Synthesized BAC-based probes with an average size of ~250 bp were previously used for development of the CATCH-Seq procedure by in-solution hybridization [13, 14]. Following a protocol presented in the previous CATCH-seq, but undergoing two rounds of hybridization capture (see Methods), we referred to this strategy as dCATCH-Seq. We first compared the target efficiency of dCATCH-Seq versus a single round of capture using the standard CATCH-Seq method. One region was captured with a pool of probes using a BAC DNA as a template covering ~248.6 kb on chromosome 11 (Fig. 1a). For both methods, we captured with at least five technical replicates along with 2 × 100 bp paired-end sequencing. A total of 170,700 and 140,200 reads on average were obtained across technical replicates for the single and double hybridizations, respectively, and on average 96.3% of the reads were mapped to the reference genome (Additional file 1: Table S1). The on target rate (target specificity) was calculated for all replicates by dividing the number of mapped reads within the target coordinates by the total number of mapped reads. On target rates were 92.2% ± 2.65% and 37.2% ± 4.34% (mean ± s.d.) for the double and single hybridization approaches, respectively (Fig. 1b). We then merged the data across technical replicates for each approach, which resulted in over 100X coverage across the intended region (Table 1). The target specificity was more than doubled for dCATCH-Seq with each approach having identical sequencing depth. Using read depth covered within 200 bp non-overlapping windows across the target site, dCATCH-Seq had an extended proportion of read bins covered with higher depth compared to single hybridization (Fig. 1c), regardless of the fraction of repeats (repeat-masked or not).

**Table 1:** Summary of merged sequencing data in a comparison between dCATCH-Seq and CATCH-Seq

Experiment	Total reads	Aligned reads	Mean PCR duplicates (%)	Aligned reads on target	On-target rate (%)	Uniquely aligned reads^a^	Uniquely aligned reads on target^a^	On-target rate (%)^a^	Mean coverage on target
dCATCH-Seq CATCH-Seq	853,358 841,154	414,965 802,931	47.4 2.8	382,625 291,808	92.21 36.34	406,331 762,445	379,084 289,558	93.29 37.98	145.98 110.18

^a^Uniqueness and relevant statistics are calculated from reads with mapping quality (MAPQ) ≥ 20

We next addressed whether two rounds of capture could introduce greater bias within the targeted intervals. We normalized read coverage between the two methods by calculating the read depth per 200 bp window divided by the total number of mapped reads within the entire target (with log_2_ transformation) to measure uniformity of coverage across the target in both approaches. Both approaches were highly concordant (R^2^ = 0.93, *P* < 1X10^−16^, Fig. 1d), which suggests no further regional bias was introduced from an additional capture in the dCATCH-Seq method.

We further conducted a comparative analysis between these two approaches in terms of the accuracy in the identification of genetic variants. In total, 259 single nucleotide variants (SNVs) were identified by two methods, where 253 were known single nucleotide polymorphisms (SNPs). Among them, 115 were homozygous SNVs. All were concordantly determined by both methods. Except for three discordantly identified SNVs located in the simple repeat (polyadenine) regions (Additional file 2: Figure S1), the remaining 144 heterozygous SNVs are concordantly called from the dCATCH-Seq data and the CATCH-Seq data. Therefore, an estimation of the accuracy rate in variant calls for dCATCH-Seq was ~99.2% relative to the single hybridization approach. We also presented a comparison of the allelic depth for 141 heterozygous SNVs called from both methods. As Fig. 1e shows, the dCATCH-Seq approach did not introduce allelic bias relative to the single hybridization.

We also compared the Indel calls between the two methods. Of 32 Indels identified, 30 (~93.8%) showed a concordance of genotype calls (Additional file 1: Table S2). The remaining two discordantly called Indels were identified as heterozygous from the dCATCH-Seq data, while as homozygous from the CATCH-Seq data (Additional file 2: Figure S2). Using independent CATCH-seq assays in two replicates, we further validated that these two Indels are heterozygous insertions (Additional file 2: Figure S3).

### Double hybridization capture on the MHC region

**Fig. 2:**
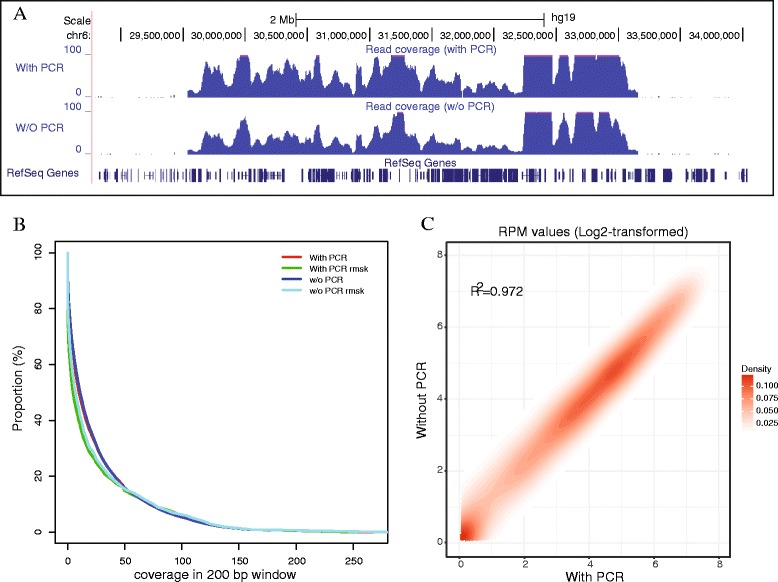
Double capture and sequencing of the MHC region in K562 cells. **a** Wiggle plot showing the capture of the human MHC region with a size of ~3.5 Mb (chr6:29,651,300–33,160,000, hg19) under two conditions (with PCR amplification or not). **b** Plot of the proportion of reads against the read depth based on coverage in 200-bp non-overlapping windows across the MHC region for the two conditions. Rmsk: repeat masker. **c** Density plot showing the comparison of read depth normalized by using the log2-transformed RPM value between two conditions

We next tested two conditions with or without PCR amplification after first round of capture using a synthesized probe set on a ~3.5 Mb continuous MHC region that covers the three major classes of MHC molecules with DNA libraries prepared from K562 cells (Fig. 2a). The sequencing data showed that both conditions were capable of capturing the entire MHC region with identically high target specificity (92.7%) and alignment rate (97%, Table 2). Comparative analyses based on both raw read coverage and normalized read coverage binned into 200 bp windows also showed that the two conditions exhibited high concordance (R^2^ = 0.972, *P* < 1X10^−16^, Fig. 2b and c).

**Table 2:** Summary of the MHC regional capture using dCATCH-Seq in three cell genomes

Cell	Condition^a^	Total reads	Aligned reads	Alignment rate (%)	PCR duplicate rate (%)	Aligned reads on target	On-target rate (%)	Uniquely aligned reads on target^b^	On-target rate (%)^b^
K562	With PCR	2,882,056	2,744,145	97.79	2.67	2,503,434	91.23	2,342,653	92.77
K562	Without PCR	1,922,388	1,795,403	97.67	4.43	1,633,616	90.99	1,528,150	92.71
GM12878	Without PCR	4,243,904	3,836,649	98.44	8.39	3,492,283	91.02	3,228,811	92.28
U937	Without PCR	1,489,332	1,244,031	95.96	13.63	1,033,606	83.09	983,376	86.38

^a^Conditions are defined based on with or without PCR amplification of DNA library after the first round of capture

^b^Uniqueness and relevant statistics are calculated from reads with MAPQ ≥20

By comparing the efficiency of detecting structural variants (SVs) between the two conditions, we also found a similar performance for both (Additional file 2: Figure S4). In comparison with previously reported SVs from a whole-genome sequencing study in the same cell line [18], both methods could detect the majority of reported SVs across the MHC region (Additional file 2: Figure S4). In the present study, we also discovered two novel deletions in K562 cell genome. One was a homozygous deletion spanning ~2.5 kb in size (Additional file 2: Figure S5A, B), and the other was an ~800 bp heterozygous deletion (Additional file 2: Figure S5C, D). Both deletions were confirmed by the PCR using primers that flanked the respective deletions (Additional file 2: Figure S5B and S5D). Altogether, our results suggest that PCR amplification of DNA library after the first hybridization capture is an optional step.

**Fig. 3:**
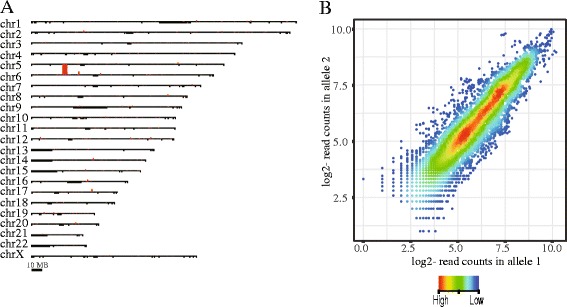
Double capture and sequencing of the MHC region in the GM12878 genome. **a** Genome-wide distribution of read coverage in GM12878 genome for dCATCH-Seq. Red bars shown above chromosomes represent the read coverage in 5 kb windows. Black bars shown below chromosomes represent the gap (unassembled) regions in reference human genome. **b** Scatterplot of read counts (log2-transformed) for both alleles called among heterozygous variants called within the MHC region. Colors in a rainbow mode represent the density of heterozygous variants

We again performed dCATCH-Seq on the human MHC region with no PCR amplification after the first hybridization capture with DNA libraries prepared from GM12878 and U937 cells. Similar to results from K562 cells, we reproducibly captured the entire MHC region with a target specificity of ~90% (Fig. 3a, Additional file 2: Figure S6 and Table 2) in these two cell genomes. A comparative analysis of the allelic depth for 8913 heterozygous SNVs identified across the MHC region for GM12878 cell lines showed that both alleles were almost equally captured (Fig. 3b). The result was consistent with a previous study showing that probes in long length (e.g., > 150 bases) are tolerant to polymorphisms [19]. We also observed a similar result for the allelic depth of 7116 heterozygous SNVs called in U937 cells (Additional file 2: Figure S7). We further evaluated the accuracy of variant calls on the MHC region. Compared to microarray genotyping data, there were 277 SNPs within the sequenced target that overlapped with microarray probes. Among them, three SNPs (two were heterozygous and one homozygous in our dCATCH-Seq data) showed the discordance with microarray calls. Overall, the genotyping accuracy was ~99% in agreement between the dCATCH-Seq approach and microarray data, which is consistent with our previous observations shown above.

We successfully obtained genotypic information for 26 HLA genes in two cell genomes and determined HLA typing with dCATCH-Seq data (Additional file 1: Table S3). To evaluate the accuracy of HLA genotyping, we compared our results with a previous report in the GM12878 genome [20]. The results showed that among 15 typed HLA genes, 13 genes were concordantly typed, especially the five classic HLA class I (HLA-B, C) and HLA class II (HLA-DQA1, DQB1, DRB1) genes that were further validated in this previous study [20]. Taken together, regardless of selected genomic features and size, dCATCH-Seq is a robust method to capture any DNA sequences with extremely high target specificity with no substantial diminution of library diversity and introduced bias.

### Double capture followed by bisulfite sequencing

Targeted bisulfite sequencing technology is a robust approach to capture regional DNA methylation levels [21]. To demonstrate the utility of the double capture approach coupled with bisulfite sequencing, we used the dCATCH-Seq procedure on the same targets shown above followed by bisulfite conversion and sequencing. Due to the severe effect of library degradation by bisulfite treatment [22], additional PCR cycles are required to amplify the bisulfite-treated DNA library (e.g., > 20 cycles), which results in high PCR duplication rate in the bisulfite sequencing data. We first determined whether the PCR amplification would introduce bias in quantifying DNA methylation levels. We compared the DNA methylation levels based on a read coverage threshold of at least 10 between two conditions (deduplication of PCR amplified reads or not). The sequencing results for both conditions tested in two cells were summarized in Additional file 1: Table S4. A comparative analysis showed a high concordance rate of CpG methylation levels between the two conditions across both the chr11 region (*n* = 2666, R^2^ = 0.99, *P* < 1X10^−16^, Additional file 2: Figure S8A) and the MHC region (*n* = 3942, R^2^ = 0.97, *P* < 1X10^−16^, Additional file 2: Figure S8C). Similar patterns were found when comparing non-CpG methylation calls in either CHG or CHH dinucleotide context on both regions (Additional file 2: Figure S8B and Figure S8D).

**Fig. 4:**
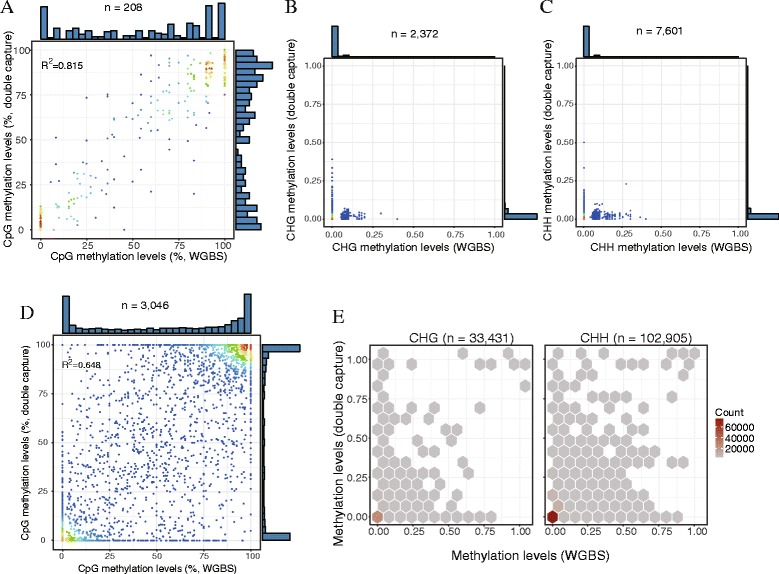
Identification of DNA methylation levels by double capture followed by bisulfite sequencing. Correlation of DNA methylation levels at a region on chr11 (**a**-**c**) and on the MHC region (**d**-**e**) identified by dCATCH-BSseq (y-axis) and whole-genome bisulfite-sequencing (WGBS, x-axis) techniques in the CpG, CHG and CHH dinucleotide contexts, respectively. Colorful dots shown in A-D denote the density of DNA methylation sites, and histograms shown outside the axes denote the distribution of DNA methylation levels

Lastly, we compared DNA methylation profiles between dCATCH-Seq and whole-genome bisulfite sequencing (WGBS) data in the same cell lines. We used the targeted region on chr11 as an example, and the result showed a high concordance (*n* = 208, R^2^ = 0.82, *P* < 1X10^−16^, Fig. 4a) of CpG methylation levels detected by both methods. Similarly, both determined a low DNA methylation profiling of the CHG and CHH sites (Fig. 4b and c), which are predominantly found present in brain tissue and embryos [23–25]. A similar pattern with a slightly less correlation (R^2^ = 0.65, *P* < 1X10^−16^, Fig. 4d and e) was observed across the MHC region in GM12878 cells. Altogether, we conclude that the dCATCH-Seq method could also be used to investigate DNA methylation profiles across any genomic regions of interest, and additional PCR cycles did not introduce bias in methylation calls.

## Discussion

For efficient targeted sequencing for research and diagnostic purposes, capture of DNA or RNA sequence with high specificity is critical. In this study, we presented an improved targeted DNA sequencing approach by using a strategy of two rounds of hybrid selection. Relative to our previous and other reports about the target specificity [9–15], the present approach significantly increases the efficiency of target enrichment. A previous study also used a similar strategy, but captured small, non-continuous and less complex genomic intervals (<20 kb) with array-based synthesized probes [26]. We also demonstrate high accuracy and reproducibility of the dCATCH-Seq method in DNA methylation quantitative analyses.

Owing to different design and purpose on capturing genomic regions, a comparison of the dCATCH-Seq method with other target enrichment platforms, e.g. exome enrichment, is generally inappropriate. Given that the enrichment technique for some commercial target enrichment platforms [7], including TruSeq (Illumina), SureSelect (Agilent), SeqCap (Roche NimbleGen), xGen (IDT), MYbaits (MYcroarray) and FleXelect (FlexGen), is similar with the dCATCH-Seq, all based on the hybrid capture, we speculate that the strategy of two-round hybridization approach might be plausible for these hybridization-based target enrichment platforms regardless of target size.

Capture of the entire MHC region by dCATCH-Seq with high efficiency is one of the most promising applications in this study. Because this region is broadly associated with numerous diseases, including cancers [27–29], autoimmune [30–32] and infectious diseases [33], etc. Diverse genetic interactions among MHC, Killer-cell Immunoglobulin-like Receptors (KIRs) and T-cell Receptors (TCRs) are also reported in human populations [34–36].

Several additional efforts could be taken to extend the application of our method with other techniques, such as targeted RNA-seq [37], single cell targeted sequencing [38], hybrid capture of viral or transgene integration sites in host genomes [39], enrichment of the human ancient DNA extracted from archaeological samples (e.g., bones) [40], cross-species DNA capture of ultraconserved elements (UCEs) or mitochondrial genomes for phylogenomic studies [41–43]. Conversely, the double capture approach is probably also useful for negative selection to remove unwanted molecules or by-products, such as contaminating DNA within a sample. For example, it is possible to apply the double capture approach to maximally eliminate the rRNA molecules for total RNA-seq [44]. Likewise, because there are approximately 30–50% of sequenced reads mapped to the mitochondrial genome for transposase-accessible chromatin with sequencing (ATAC-seq) technology [45, 46], the double capture strategy could be probably applicable to remove the mitochondrial DNA sequence.

Finally, some limitations remain in this study. The first limitation is the unequal coverage depth across targeted regions, which is a common issue for the target enrichment methods [7]. Two potential factors could explain this issue. One factor might be due to the usage of blocking reagents, a common and necessary strategy to reduce the background signal for hybridization-based assays. The other factor could be due to the inherent DNA sequences, including G + C content, number and types of repetitive elements across targeted regions. The second limitation is the applicability and efficiency of the double-capture strategy for low-input DNA samples, particularly when the input DNAs down to the levels of tens of nanogram. Because the low amount of DNA input will substantially increase the PCR duplicates in sequencing data and subsequently reduce the superiority for the double-capture approach. Such trade-off between PCR duplicate rates and DNA amount has also been reported in previous target enrichment method [47].

## Conclusions

In conclusion, the dCATCH-Seq approach is a powerful and scalable approach to interrogate genetic and epigenetic features on genomic regions of interest, and has the potential to be further combined with other functional genomics approaches.

## Methods

### Cell culture and DNA extraction

GM12878 B-lymphoblast, K562 (a generous gift from Dr. Myer’s lab at HudsonAlpha Institute) and U937 cell lines (purchased from ATCC, cat. no. CRL-1593.2) were cultured in RPMI-1640 medium, supplemented with 10% fetal bovine serum (FBS), 2 mM L-glutamine and 1% penicillin-streptomycin in a 37 °C incubator with 5% CO_2_. Cells were collected when grown to ~90% confluence. Following three PBS washes, genomic DNA was extracted using proteinase K and phenol-chloroform method. Genomic DNA concentration was determined by Qubit fluorometer (ThermoFisher).

### Enrichment of targets by two rounds of hybridization

All BAC-based probes across intended regions were obtained from Ubiquity Genomics, Inc. DNA library construction, target capture and enrichment were previously described [13] with a few modifications. Briefly, input DNA quantities of 1 μg and 3 μg were used for standard and bisulfite converted dCATCH-Seq approaches, respectively. DNAs were sheared to ~250 bp in size using the Covaris-S220, followed by end-repair, dA-tailing and paired-end Illumina adapter (methylated adapters used for bisulfite sequencing) ligation using NEBNext reagents or Biodynami NGS DNA library prep kit. For the first capture, a hybridization reaction was assembled containing 1× library DNA, 30× human Cot-1 DNA (American Genetics), 2500× BAC-derived DNA probes, and 1× hybridization buffer and denatured at 95 °C for 5 min followed by incubation of the reaction at 65 °C for ~48 h. The first capture was performed according to our previous protocol with streptavidin-coated Dynabeads (ThermoFisher), that were washed once at room temperature for 10 min in 1× SSC with 0.1% SDS and twice at 65 °C for 10 min in 0.1× SSC with 0.1% SDS. After the first capture, an optional PCR enrichment step of the captured library was included for the dCATCH-Seq method, with 2X KAPA HiFi master mix (KAPA Biosystems) under the PCR conditions: 45 s at 98 °C; ten cycles of 15 s at 98 °C, 30 s at 60 °C, and 45 s at 72 °C. The hybridization conditions for the second capture were identical with the first, except for incubation of the hybridization reaction at 65 °C for ~24 h, and the captured samples were washed twice at room temperature by 10 min in 1× SSC with 0.1% SDS and three times at 65 °C by 10 min in 0.1× SSC with 0.1% SDS. Finally, DNA was amplified by PCR using indexed primer pairs using the same PCR conditions as above. For the dCATCH-Seq bisulfite sequencing approach, the captured DNA library was bisulfite converted using the EpiTect Bisulfite kit (Qiagen) according to the manufacturer’s instructions for small quantities of fragmented DNA. The bisulfite-treated DNA was amplified by using 5 U Platinum *Taq* DNA Polymerase (ThermoFisher) under the PCR conditions: 98 °C for 1 min, followed by 20–26 cycles of (95 °C for 30 s and 62 °C for 3 min). We confirmed the amplification and correct product size range by gel electrophoresis on a 1.7% agarose gel. Amplified libraries were purified with SPRI beads, and library sizes determined by Agilent Bioanalysis DNA high sensitivity (Agilent), and library concentrations quantified by KAPA Quant kit for Illumina (KAPA Biosystems). Libraries were sequenced according to standard Illumina protocol on MiSeq or HiSeq 2500 sequencers.

### Public data collection

We collected (1) microarray genotyping data with two replicates (sample ID: GSM1028244 and GSM1028245) for the U937 cell line, (2) WGBS data for K562 (sample ID: GSE86747) and GM12878 cell lines (sample ID: GSM1002650), and (3) SVs data from a whole-genome sequencing study for K562 cells [18].

### Data processing and statistics

Reads were demuxed based on their index sequence at the Genomic Services Laboratory at HudsonAlpha. After removal of adapter sequences, low-quality reads, and trimmed reads that were shorter than 20 bp by using cutadapt (v1.3.1), filtered reads were aligned with bowtie2 (version 2.1.0) [48] to the human hg19 reference genome. Reads were re-aligned, recalibrated and SNVs and Indels were called using the GATK toolkit (version 3.3) [49]. Variants were filtered for quality as previously described [11]. Briefly, we filtered out variants as follows: (1) mapping quality score < 20; (2) ≥ 3 SNPs detected within 10 bp distance; (3) variant confidence/quality by depth < 2; (4) strand bias score > 50; (5) genotype score < 15; (6) read depth < 10. The reads per million (RPM) was calculated as the read counts per 200-bp non-overlapping window aligned on the target region divided by per million reads scaling factor. Read mapping was visualized in the Integrative Genomics Viewer (IGV) software [50]. With BAM files as input, HLA genes were typed using the SOAP-HLA [15] with default parameters. Structural variants were called by using Pindel [51] with ≥4 supporting reads and ≥100 bp in size. The sequencing data from the bisulfite protocol with dCATCH-Seq and WGBS were aligned and DNA methylation levels at CpGs and non-CpGs (CHG and CHH dinucleotides) were estimated by using bismark (version v0.14.1) with default parameters [52]. CpGs, CHGs or CHHs with at least 10X coverage were retained for comparative analyses. Except for relevant programs described, all other bioinformatics analyses were implemented using customized Perl scripts and R programming. All customized codes are available upon request.

### Verification of structural variation

Primer pairs were designed (Additional file 1: Table S5 in and Additional file 2: Figure S5) for each SV by using 200-bp sequences flanking both sides of the deletion region. PCR products were amplified with extracted genomic DNA templates from cells with 5 U NEB *Taq* DNA Polymerase under the PCR conditions: 95 °C for 3 min, followed by 30 cycles of 95 °C for 30 s, 55 °C for 30 s and 72 °C for 45 s. PCR products were run on 2% agarose gels.

## Additional files

Additional file 1: Table S1. Summary of targeted sequencing data in multiple replicates for dCATCH-Seq and CATCH-Seq. **Table S2.** Comparison of Indel calls between dCATCH-Seq and CATCH-Seq. **Table S3.** HLA gene typing for dCATCH-Seq. **Table S4.** Summary of bisulfite dCATCH-Seq data compared between two conditions. **Table S5**: List of PCR primers for verifying SVs. (XLSX 19 kb)

Additional file 2: Figure S1 and S2. Visualization of reads mapping to three SNVs (**S1**) and two Indels (**S2**) called for dCATCH-Seq (left panel) and CATCH-Seq (right panel) methods. Red-colored arrows show the location of the discordantly called SNVs (**S1**) and Indels (**S2**). **Figure S3.** Visualization of reads mapping to two Indels (A and B) from an independent CATCH-Seq assay in two replicates. Red-colored arrows, the location of the confirmed Indels. **Figure S4.** SVs in K562 cells. The upper panels are SVs detected by conditions (with or without PCR) after the first capture. The lower panels are SVs identified by a previous study. **Figure S5.** Two novel deletions in K562 cells. PCR primer pairs for verifying a homozygous deletion (A) and a heterozygous deletion (C), and the agarose gel electrophoresis results (B and D). P, N and M denote the positive, negative bands and DNA marker, respectively. Due to difficulty in design of the specific primers, there is a second (non-specific) band for the N2 lane. **Figure S6.** Genome-wide read coverage in U937 cell genome using the dCATCH-Seq. Red bars represent the read depth in 5 kb windows. Black bars mean the gap (unassembled) regions in the reference human genome. **Figure S7.** Scatterplot showing read counts (log2-transformed) on two alleles for heterozygous variants across the MHC region in U937 cell genome. Colors in a rainbow mode represent the density of heterozygous variants. **Figure S8.** Comparison of DNA methylation levels at a region on chr11 (A-B) and on the MHC region (C-D) calculated by read counts with (x-axis) or without (y-axis) removal of PCR duplicates in a CpG (left panel) or non-CpG (right panel) dinucleotide context. Histograms (A and C) represent the distribution of CpG methylation levels corresponding to x- and y-axes, respectively. (ZIP 8432 kb)

## Abbreviations

dCATCH-Seq: Double CATCH-Seq
KIR: Killer-cell Immunoglobulin-like Receptor
MHC: Major histocompatibility complex
SNP: Single nucleotide polymorphism
SNV: Single nucleotide variant
SV: Structural variant
TCR: T-cell Receptor; RPM: reads per million
WGBS: Whole-genome bisulfite sequencing
