# Supplementary figures and images for: dCATCH-Seq: improved sequencing of large continuous genomic targets with double-hybridization
Source: BMC Genomics. 2017 Oct 23;18:811. doi: 10.1186/s12864-017-4159-7 (PMC5653981; doi:10.1186/s12864-017-4159-7)

A

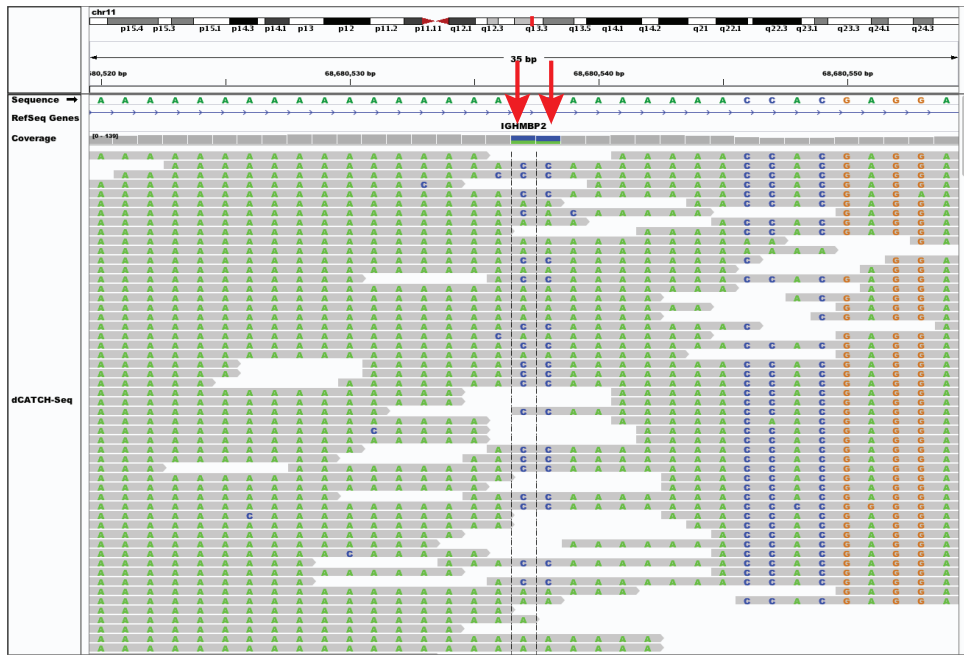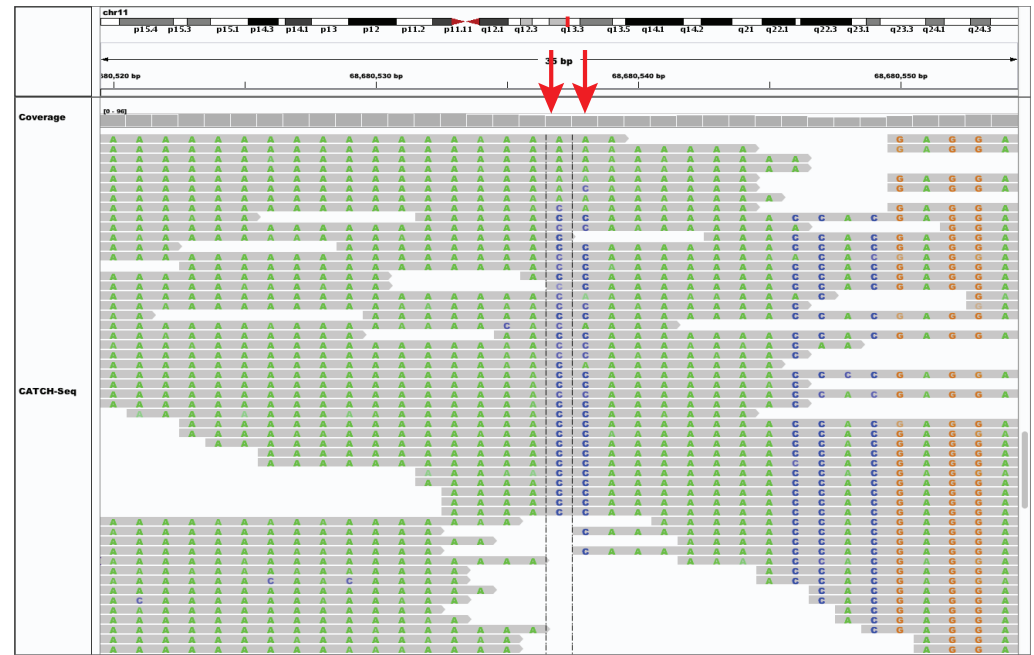

B

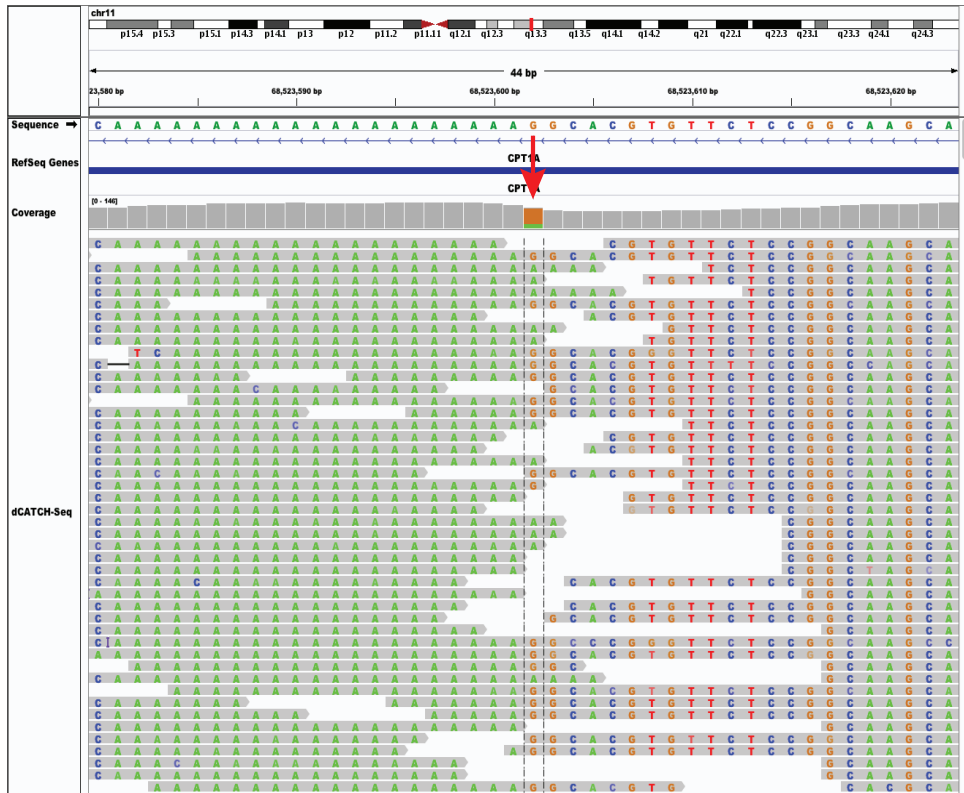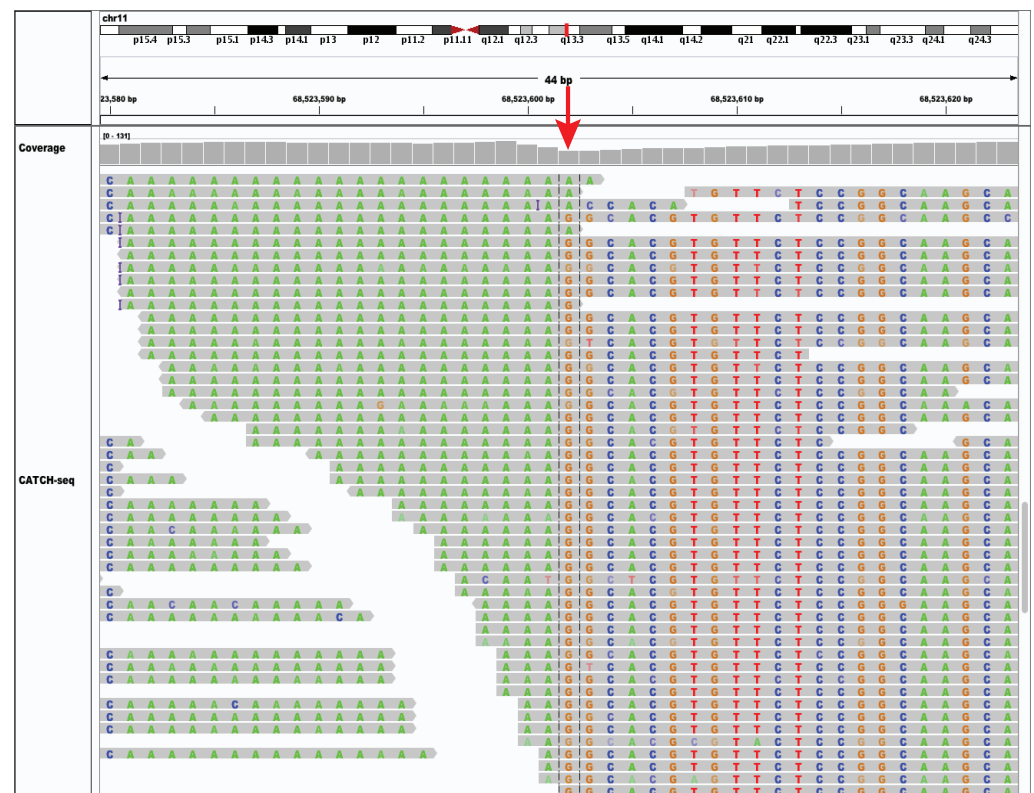

Supplement: Supplementary file 2 — Visualization of reads mapping to three SNVs (S1) and two Indels (S2) called for dCATCH-Seq (left panel) and CATCH-Seq (right panel) methods. Red-colored arrows show the location of the discordantly called SNVs (S1) and Indels (S2). Figure S3. Visualization of reads mapping to two Indels (A and B) from an independent CATCH-Seq assay in two replicates. Red-colored arrows, the location of the confirmed Indels. Figure S4. SVs in K562 cells. The upper panels are SVs detected by conditions (with or without PCR) after the first capture. The lower panels are SVs identified by a previous study. Figure S5. Two novel deletions in K562 cells. PCR primer pairs for verifying a homozygous deletion (A) and a heterozygous deletion (C), and the agarose gel electrophoresis results (B and D). P, N and M denote the positive, negative bands and DNA marker, respectively. Due to difficulty in design of the specific primers, there is a second (non-specific) band for the N2 lane. Figure S6. Genome-wide read coverage in U937 cell genome using the dCATCH-Seq. Red bars represent the read depth in 5 kb windows. Black bars mean the gap (unassembled) regions in the reference human genome. Figure S7. Scatterplot showing read counts (log2-transformed) on two alleles for heterozygous variants across the MHC region in U937 cell genome. Colors in a rainbow mode represent the density of heterozygous variants. Figure S8. Comparison of DNA methylation levels at a region on chr11 (A-B) and on the MHC region (C-D) calculated by read counts with (x-axis) or without (y-axis) removal of PCR duplicates in a CpG (left panel) or non-CpG (right panel) dinucleotide context. Histograms (A and C) represent the distribution of CpG methylation levels corresponding to x- and y-axes, respectively. (ZIP 8432 kb) [file 12864_2017_4159_MOESM2_ESM.zip › Figure S1_Additional file 2.pdf]

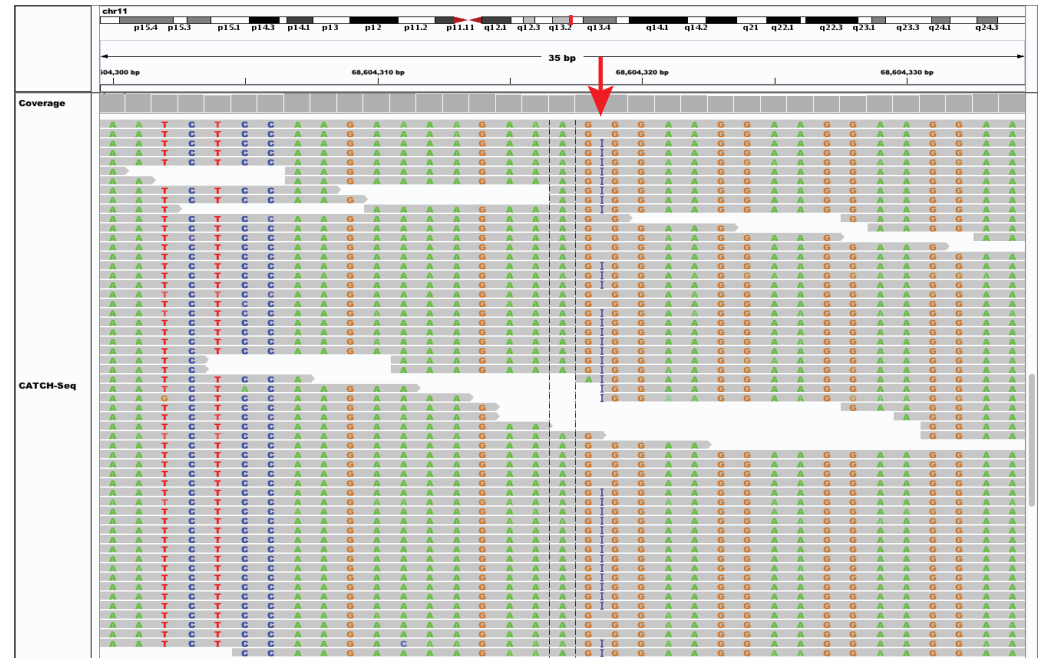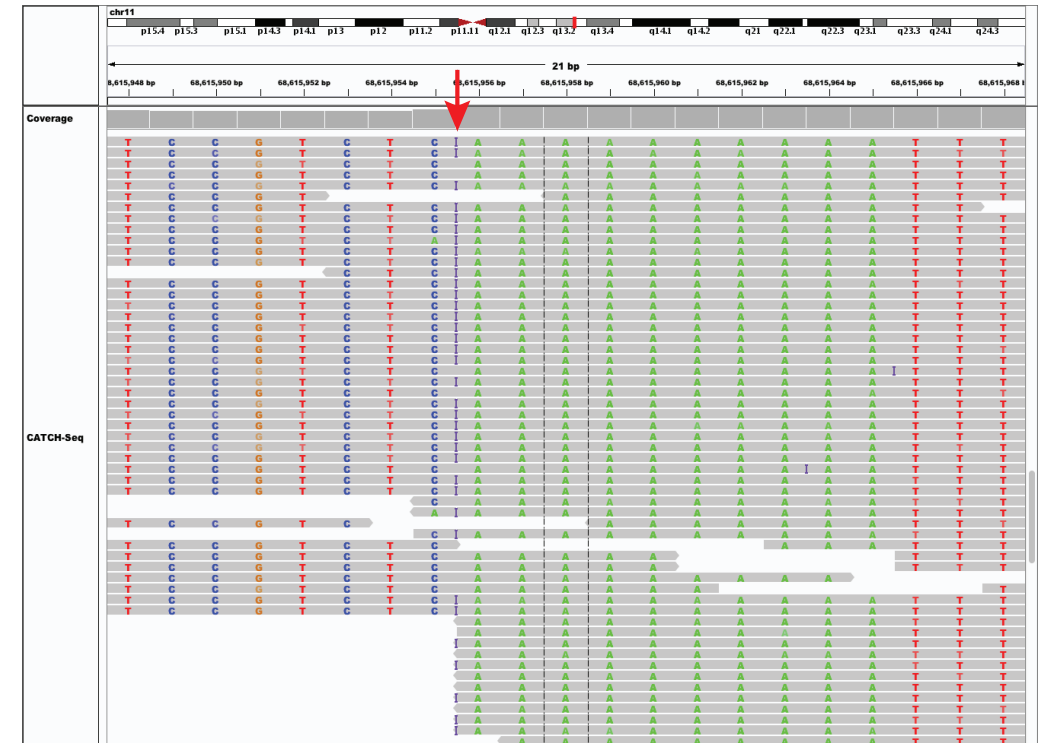

Supplement: Supplementary file 2 — Visualization of reads mapping to three SNVs (S1) and two Indels (S2) called for dCATCH-Seq (left panel) and CATCH-Seq (right panel) methods. Red-colored arrows show the location of the discordantly called SNVs (S1) and Indels (S2). Figure S3. Visualization of reads mapping to two Indels (A and B) from an independent CATCH-Seq assay in two replicates. Red-colored arrows, the location of the confirmed Indels. Figure S4. SVs in K562 cells. The upper panels are SVs detected by conditions (with or without PCR) after the first capture. The lower panels are SVs identified by a previous study. Figure S5. Two novel deletions in K562 cells. PCR primer pairs for verifying a homozygous deletion (A) and a heterozygous deletion (C), and the agarose gel electrophoresis results (B and D). P, N and M denote the positive, negative bands and DNA marker, respectively. Due to difficulty in design of the specific primers, there is a second (non-specific) band for the N2 lane. Figure S6. Genome-wide read coverage in U937 cell genome using the dCATCH-Seq. Red bars represent the read depth in 5 kb windows. Black bars mean the gap (unassembled) regions in the reference human genome. Figure S7. Scatterplot showing read counts (log2-transformed) on two alleles for heterozygous variants across the MHC region in U937 cell genome. Colors in a rainbow mode represent the density of heterozygous variants. Figure S8. Comparison of DNA methylation levels at a region on chr11 (A-B) and on the MHC region (C-D) calculated by read counts with (x-axis) or without (y-axis) removal of PCR duplicates in a CpG (left panel) or non-CpG (right panel) dinucleotide context. Histograms (A and C) represent the distribution of CpG methylation levels corresponding to x- and y-axes, respectively. (ZIP 8432 kb) [file 12864_2017_4159_MOESM2_ESM.zip › Figure S2_Additional file 2.pdf]

A

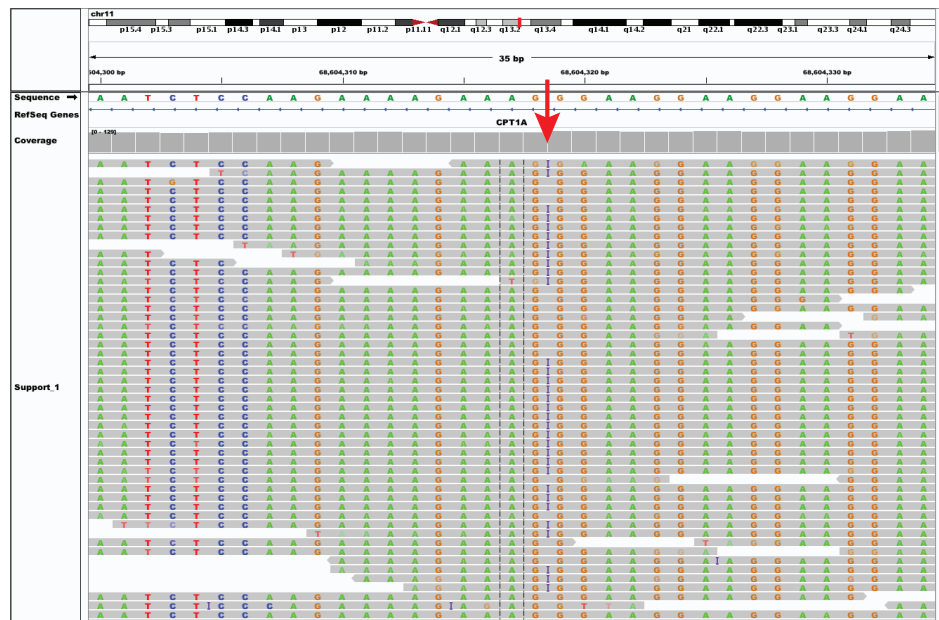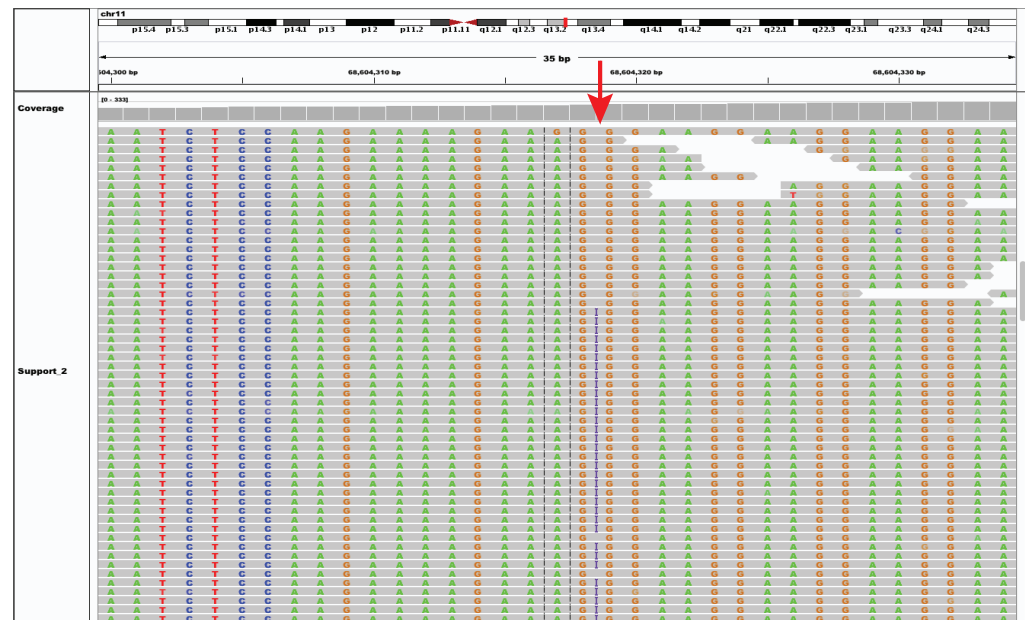

B

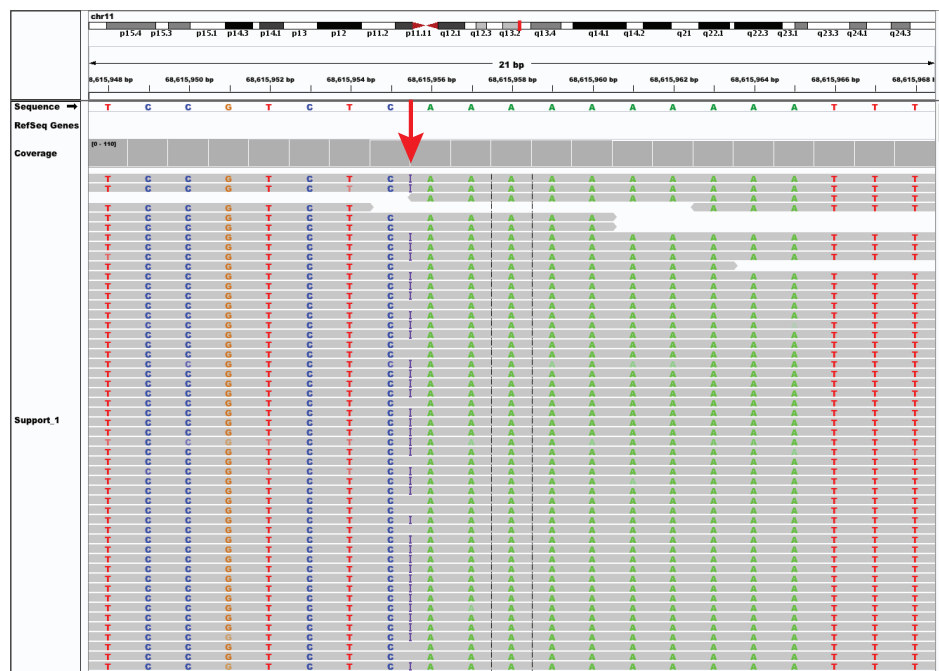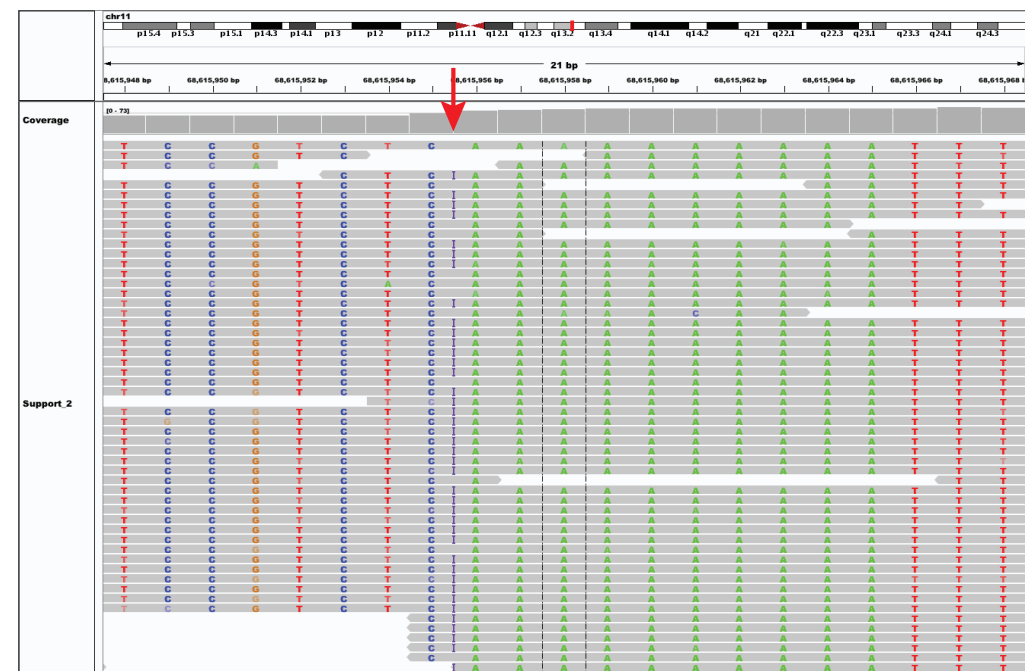

Supplement: Supplementary file 2 — Visualization of reads mapping to three SNVs (S1) and two Indels (S2) called for dCATCH-Seq (left panel) and CATCH-Seq (right panel) methods. Red-colored arrows show the location of the discordantly called SNVs (S1) and Indels (S2). Figure S3. Visualization of reads mapping to two Indels (A and B) from an independent CATCH-Seq assay in two replicates. Red-colored arrows, the location of the confirmed Indels. Figure S4. SVs in K562 cells. The upper panels are SVs detected by conditions (with or without PCR) after the first capture. The lower panels are SVs identified by a previous study. Figure S5. Two novel deletions in K562 cells. PCR primer pairs for verifying a homozygous deletion (A) and a heterozygous deletion (C), and the agarose gel electrophoresis results (B and D). P, N and M denote the positive, negative bands and DNA marker, respectively. Due to difficulty in design of the specific primers, there is a second (non-specific) band for the N2 lane. Figure S6. Genome-wide read coverage in U937 cell genome using the dCATCH-Seq. Red bars represent the read depth in 5 kb windows. Black bars mean the gap (unassembled) regions in the reference human genome. Figure S7. Scatterplot showing read counts (log2-transformed) on two alleles for heterozygous variants across the MHC region in U937 cell genome. Colors in a rainbow mode represent the density of heterozygous variants. Figure S8. Comparison of DNA methylation levels at a region on chr11 (A-B) and on the MHC region (C-D) calculated by read counts with (x-axis) or without (y-axis) removal of PCR duplicates in a CpG (left panel) or non-CpG (right panel) dinucleotide context. Histograms (A and C) represent the distribution of CpG methylation levels corresponding to x- and y-axes, respectively. (ZIP 8432 kb) [file 12864_2017_4159_MOESM2_ESM.zip › Figure S3_Additional file 2.pdf]

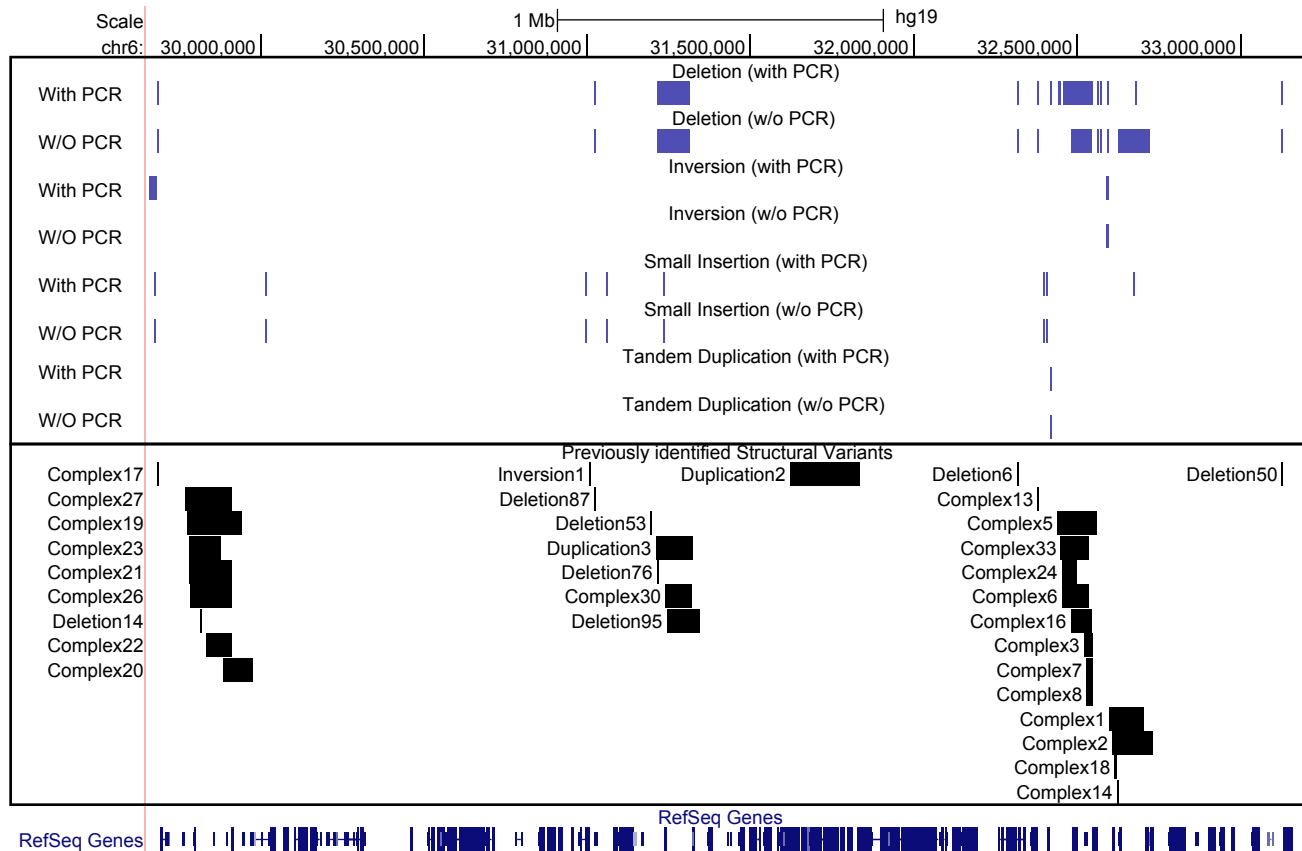

Supplement: Supplementary file 2 — Visualization of reads mapping to three SNVs (S1) and two Indels (S2) called for dCATCH-Seq (left panel) and CATCH-Seq (right panel) methods. Red-colored arrows show the location of the discordantly called SNVs (S1) and Indels (S2). Figure S3. Visualization of reads mapping to two Indels (A and B) from an independent CATCH-Seq assay in two replicates. Red-colored arrows, the location of the confirmed Indels. Figure S4. SVs in K562 cells. The upper panels are SVs detected by conditions (with or without PCR) after the first capture. The lower panels are SVs identified by a previous study. Figure S5. Two novel deletions in K562 cells. PCR primer pairs for verifying a homozygous deletion (A) and a heterozygous deletion (C), and the agarose gel electrophoresis results (B and D). P, N and M denote the positive, negative bands and DNA marker, respectively. Due to difficulty in design of the specific primers, there is a second (non-specific) band for the N2 lane. Figure S6. Genome-wide read coverage in U937 cell genome using the dCATCH-Seq. Red bars represent the read depth in 5 kb windows. Black bars mean the gap (unassembled) regions in the reference human genome. Figure S7. Scatterplot showing read counts (log2-transformed) on two alleles for heterozygous variants across the MHC region in U937 cell genome. Colors in a rainbow mode represent the density of heterozygous variants. Figure S8. Comparison of DNA methylation levels at a region on chr11 (A-B) and on the MHC region (C-D) calculated by read counts with (x-axis) or without (y-axis) removal of PCR duplicates in a CpG (left panel) or non-CpG (right panel) dinucleotide context. Histograms (A and C) represent the distribution of CpG methylation levels corresponding to x- and y-axes, respectively. (ZIP 8432 kb) [file 12864_2017_4159_MOESM2_ESM.zip › Figure S4_Additional file 2.pdf]

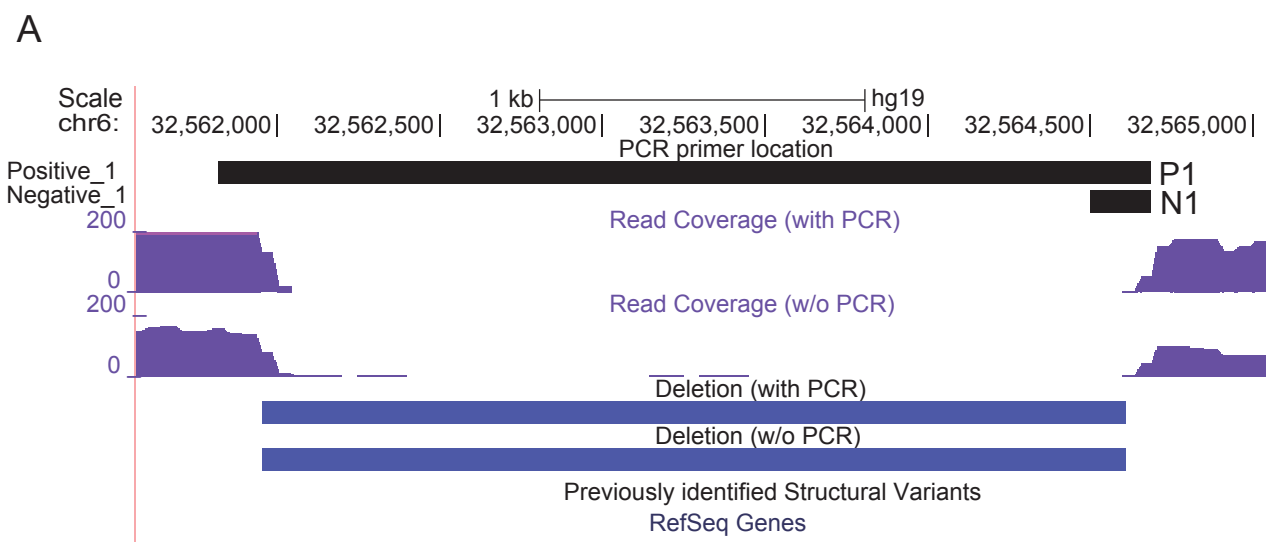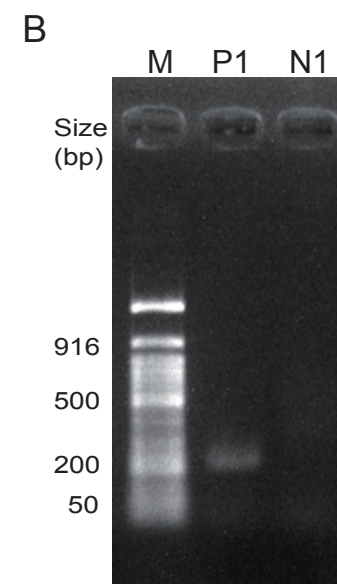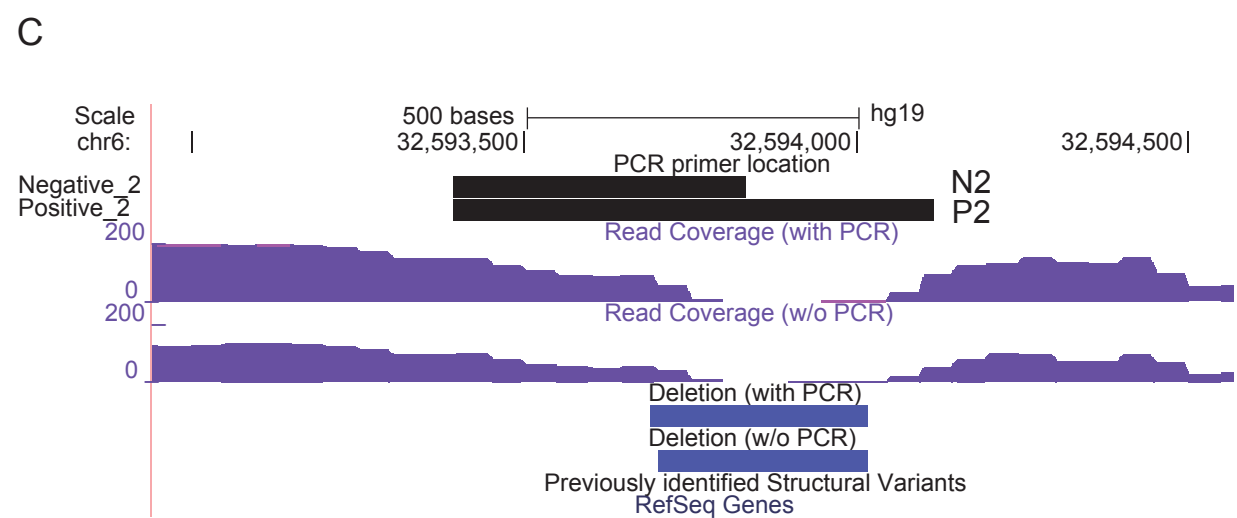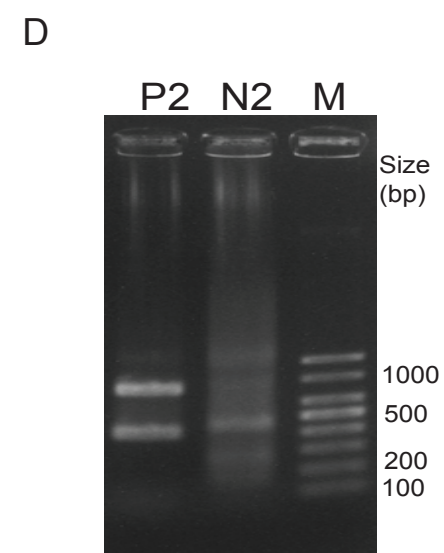

Supplement: Supplementary file 2 — Visualization of reads mapping to three SNVs (S1) and two Indels (S2) called for dCATCH-Seq (left panel) and CATCH-Seq (right panel) methods. Red-colored arrows show the location of the discordantly called SNVs (S1) and Indels (S2). Figure S3. Visualization of reads mapping to two Indels (A and B) from an independent CATCH-Seq assay in two replicates. Red-colored arrows, the location of the confirmed Indels. Figure S4. SVs in K562 cells. The upper panels are SVs detected by conditions (with or without PCR) after the first capture. The lower panels are SVs identified by a previous study. Figure S5. Two novel deletions in K562 cells. PCR primer pairs for verifying a homozygous deletion (A) and a heterozygous deletion (C), and the agarose gel electrophoresis results (B and D). P, N and M denote the positive, negative bands and DNA marker, respectively. Due to difficulty in design of the specific primers, there is a second (non-specific) band for the N2 lane. Figure S6. Genome-wide read coverage in U937 cell genome using the dCATCH-Seq. Red bars represent the read depth in 5 kb windows. Black bars mean the gap (unassembled) regions in the reference human genome. Figure S7. Scatterplot showing read counts (log2-transformed) on two alleles for heterozygous variants across the MHC region in U937 cell genome. Colors in a rainbow mode represent the density of heterozygous variants. Figure S8. Comparison of DNA methylation levels at a region on chr11 (A-B) and on the MHC region (C-D) calculated by read counts with (x-axis) or without (y-axis) removal of PCR duplicates in a CpG (left panel) or non-CpG (right panel) dinucleotide context. Histograms (A and C) represent the distribution of CpG methylation levels corresponding to x- and y-axes, respectively. (ZIP 8432 kb) [file 12864_2017_4159_MOESM2_ESM.zip › Figure S5_Additional file 2.pdf]

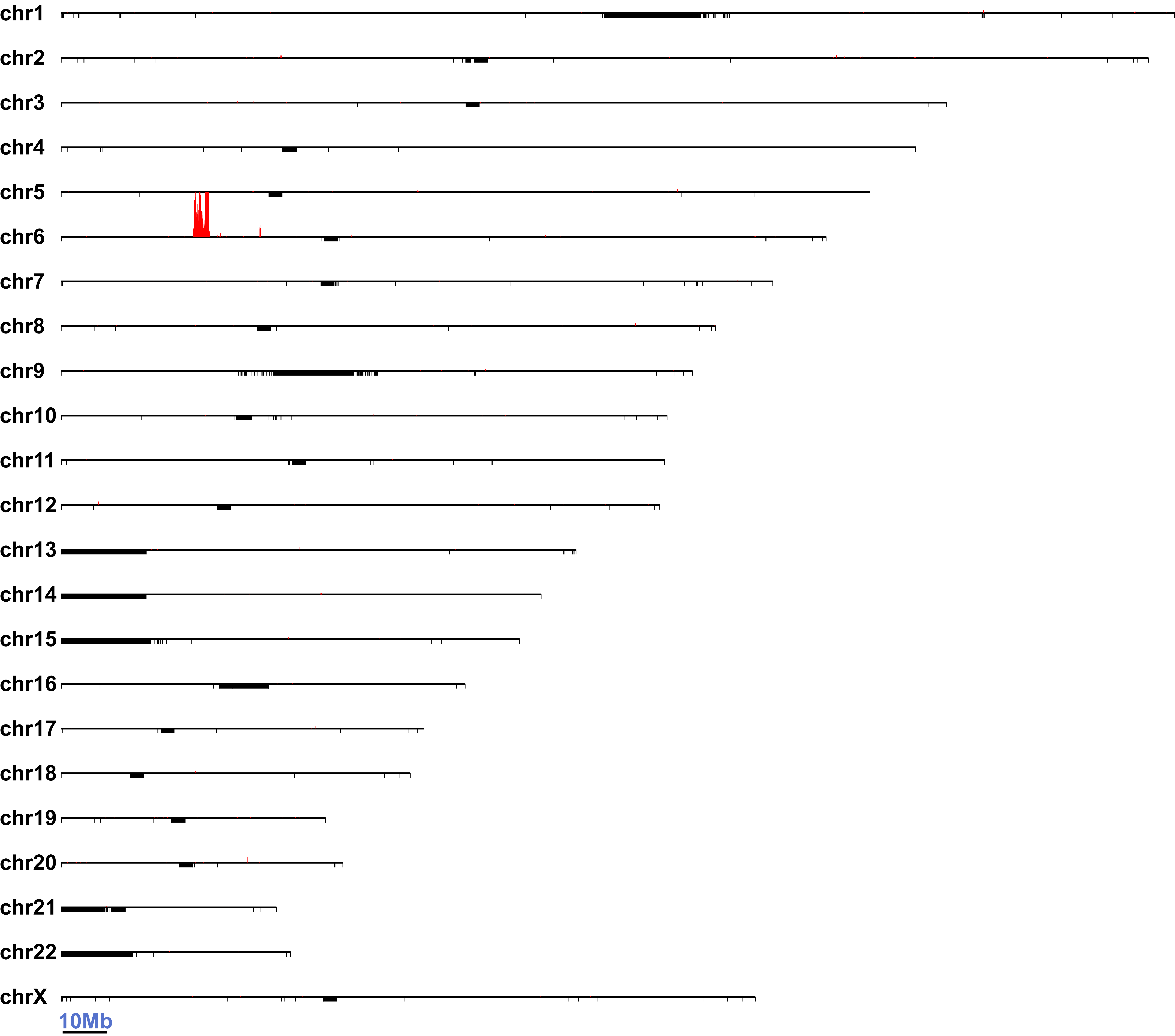

Supplement: Supplementary file 2 — Visualization of reads mapping to three SNVs (S1) and two Indels (S2) called for dCATCH-Seq (left panel) and CATCH-Seq (right panel) methods. Red-colored arrows show the location of the discordantly called SNVs (S1) and Indels (S2). Figure S3. Visualization of reads mapping to two Indels (A and B) from an independent CATCH-Seq assay in two replicates. Red-colored arrows, the location of the confirmed Indels. Figure S4. SVs in K562 cells. The upper panels are SVs detected by conditions (with or without PCR) after the first capture. The lower panels are SVs identified by a previous study. Figure S5. Two novel deletions in K562 cells. PCR primer pairs for verifying a homozygous deletion (A) and a heterozygous deletion (C), and the agarose gel electrophoresis results (B and D). P, N and M denote the positive, negative bands and DNA marker, respectively. Due to difficulty in design of the specific primers, there is a second (non-specific) band for the N2 lane. Figure S6. Genome-wide read coverage in U937 cell genome using the dCATCH-Seq. Red bars represent the read depth in 5 kb windows. Black bars mean the gap (unassembled) regions in the reference human genome. Figure S7. Scatterplot showing read counts (log2-transformed) on two alleles for heterozygous variants across the MHC region in U937 cell genome. Colors in a rainbow mode represent the density of heterozygous variants. Figure S8. Comparison of DNA methylation levels at a region on chr11 (A-B) and on the MHC region (C-D) calculated by read counts with (x-axis) or without (y-axis) removal of PCR duplicates in a CpG (left panel) or non-CpG (right panel) dinucleotide context. Histograms (A and C) represent the distribution of CpG methylation levels corresponding to x- and y-axes, respectively. (ZIP 8432 kb) [file 12864_2017_4159_MOESM2_ESM.zip › Figure S6_Additional file 2.pdf]

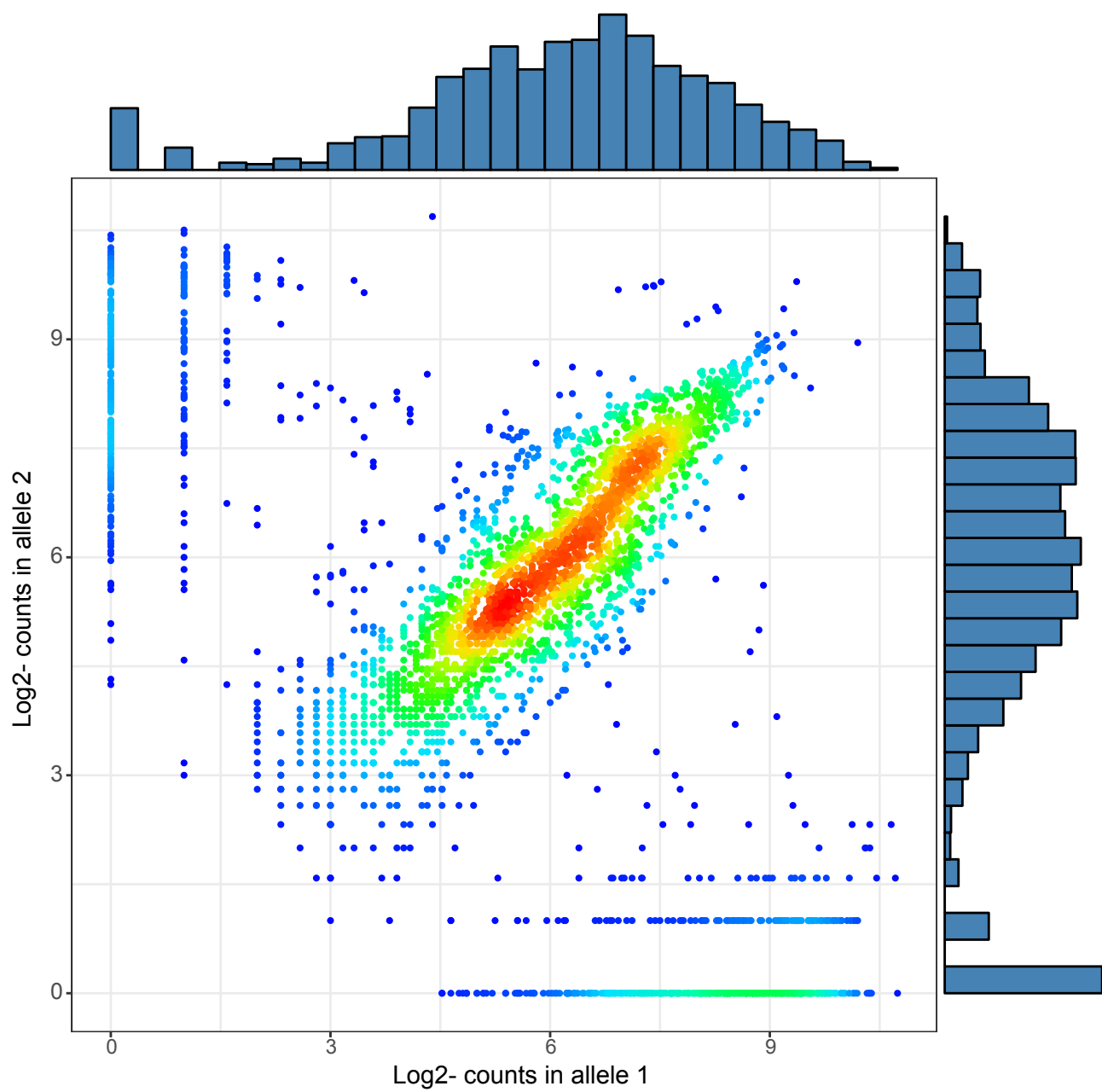

Supplement: Supplementary file 2 — Visualization of reads mapping to three SNVs (S1) and two Indels (S2) called for dCATCH-Seq (left panel) and CATCH-Seq (right panel) methods. Red-colored arrows show the location of the discordantly called SNVs (S1) and Indels (S2). Figure S3. Visualization of reads mapping to two Indels (A and B) from an independent CATCH-Seq assay in two replicates. Red-colored arrows, the location of the confirmed Indels. Figure S4. SVs in K562 cells. The upper panels are SVs detected by conditions (with or without PCR) after the first capture. The lower panels are SVs identified by a previous study. Figure S5. Two novel deletions in K562 cells. PCR primer pairs for verifying a homozygous deletion (A) and a heterozygous deletion (C), and the agarose gel electrophoresis results (B and D). P, N and M denote the positive, negative bands and DNA marker, respectively. Due to difficulty in design of the specific primers, there is a second (non-specific) band for the N2 lane. Figure S6. Genome-wide read coverage in U937 cell genome using the dCATCH-Seq. Red bars represent the read depth in 5 kb windows. Black bars mean the gap (unassembled) regions in the reference human genome. Figure S7. Scatterplot showing read counts (log2-transformed) on two alleles for heterozygous variants across the MHC region in U937 cell genome. Colors in a rainbow mode represent the density of heterozygous variants. Figure S8. Comparison of DNA methylation levels at a region on chr11 (A-B) and on the MHC region (C-D) calculated by read counts with (x-axis) or without (y-axis) removal of PCR duplicates in a CpG (left panel) or non-CpG (right panel) dinucleotide context. Histograms (A and C) represent the distribution of CpG methylation levels corresponding to x- and y-axes, respectively. (ZIP 8432 kb) [file 12864_2017_4159_MOESM2_ESM.zip › Figure S7_Additional file 2.pdf]

A

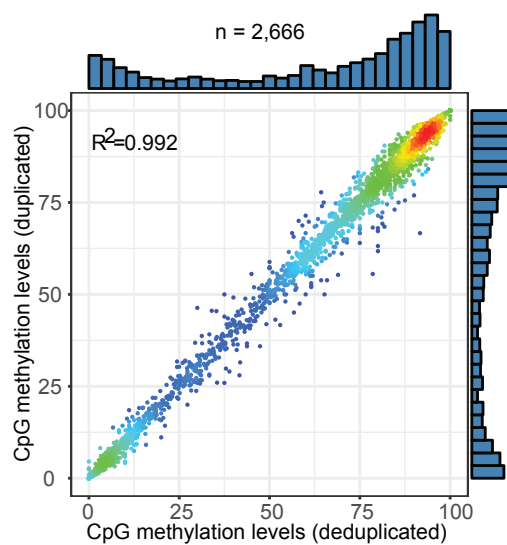

B

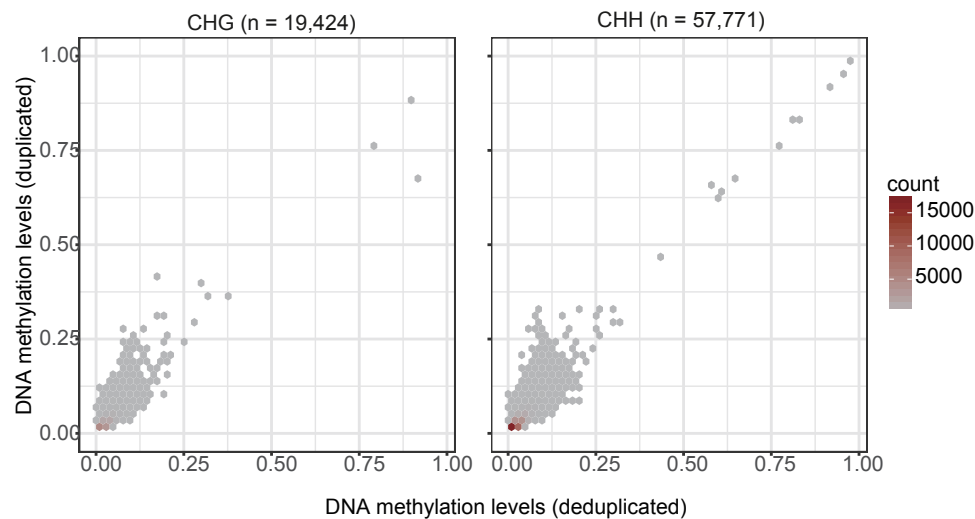

C

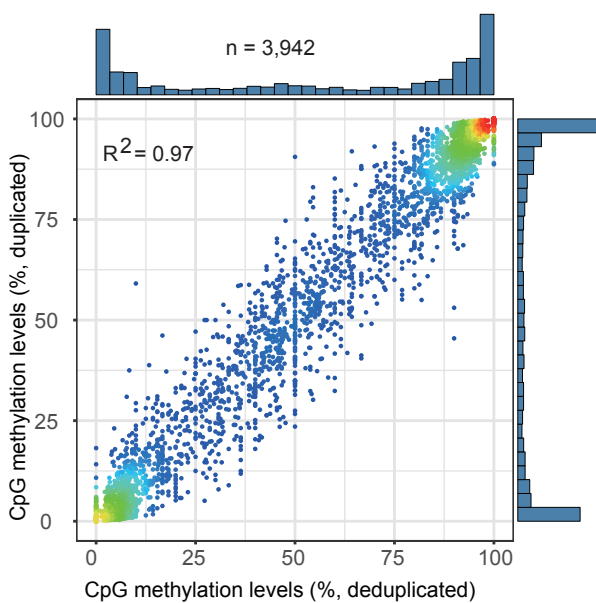

D

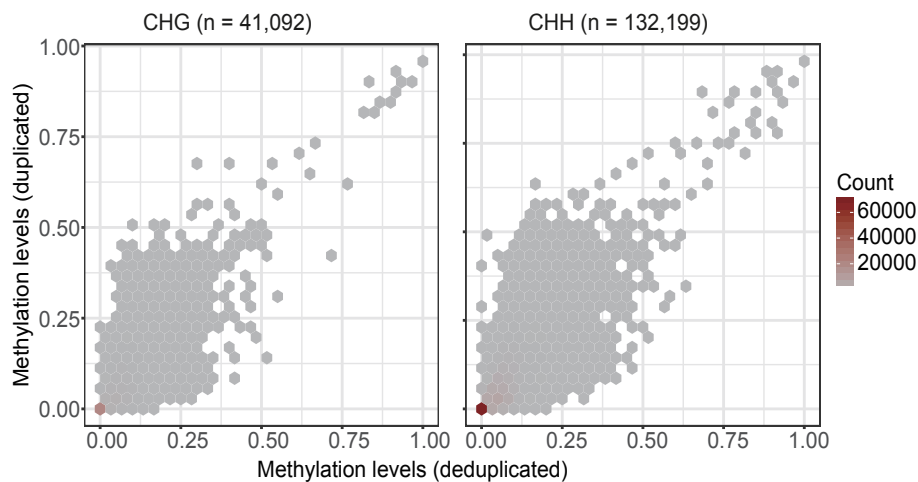

Supplement: Supplementary file 2 — Visualization of reads mapping to three SNVs (S1) and two Indels (S2) called for dCATCH-Seq (left panel) and CATCH-Seq (right panel) methods. Red-colored arrows show the location of the discordantly called SNVs (S1) and Indels (S2). Figure S3. Visualization of reads mapping to two Indels (A and B) from an independent CATCH-Seq assay in two replicates. Red-colored arrows, the location of the confirmed Indels. Figure S4. SVs in K562 cells. The upper panels are SVs detected by conditions (with or without PCR) after the first capture. The lower panels are SVs identified by a previous study. Figure S5. Two novel deletions in K562 cells. PCR primer pairs for verifying a homozygous deletion (A) and a heterozygous deletion (C), and the agarose gel electrophoresis results (B and D). P, N and M denote the positive, negative bands and DNA marker, respectively. Due to difficulty in design of the specific primers, there is a second (non-specific) band for the N2 lane. Figure S6. Genome-wide read coverage in U937 cell genome using the dCATCH-Seq. Red bars represent the read depth in 5 kb windows. Black bars mean the gap (unassembled) regions in the reference human genome. Figure S7. Scatterplot showing read counts (log2-transformed) on two alleles for heterozygous variants across the MHC region in U937 cell genome. Colors in a rainbow mode represent the density of heterozygous variants. Figure S8. Comparison of DNA methylation levels at a region on chr11 (A-B) and on the MHC region (C-D) calculated by read counts with (x-axis) or without (y-axis) removal of PCR duplicates in a CpG (left panel) or non-CpG (right panel) dinucleotide context. Histograms (A and C) represent the distribution of CpG methylation levels corresponding to x- and y-axes, respectively. (ZIP 8432 kb) [file 12864_2017_4159_MOESM2_ESM.zip › Figure S8_Additional file 2.pdf]
